# Social cognition deficits are associated with lower quality of life in cervical dystonia: A single centre study^☆^

**DOI:** 10.1016/j.prdoa.2023.100214

**Published:** 2023-08-14

**Authors:** Shameer Rafee, Ruth Monaghan, Derval McCormack, Conor Fearon, Sean O'Riordan, Michael Hutchinson, Jessica Bramham, Fiadhnait O'Keeffe

**Affiliations:** a Department of Neurology, St Vincent’s University Hospital, Ireland; b School of Medicine and Medical Sciences, University College Dublin, Ireland; c Department of Psychology, St Vincent’s University Hospital, Ireland; d School of Psychology, University College Dublin, Ireland

**Keywords:** Cervical dystonia, Social cognition, Quality of life

## Abstract

**Background and objectives:**

Patients with cervical dystonia (CD) demonstrate significant non-motor symptoms including sensory, psychiatric and cognitive features. It has been shown that the non-motor symptoms have a major influence on quality of life. Social cognition, particularly deficits in Theory of Mind (ToM), can affect the development of interpersonal relationships, understanding of social situations and can affect patient outcomes.

We used the “Faux Pas” measure of social cognition to assess ToM in patients with CD and compared this with quality of life, disease severity and psychiatric symptoms.

**Methods:**

Patients with adult-onset idiopathic isolated cervical dystonia were assessed using the “Faux Pas” questionnaire. Validated questionnaires were used to assess mood symptoms (BAI/BDI and HADS) and quality of life (CDIP-58). Disease-specific disability, motor severity and psychosocial symptoms were measured using TWSTRS2. Faux pas results were compared with published healthy control values.

**Results:**

32 participants (19 female) were included with a mean age of 57.7 years. 20 participants met criteria for excess mood symptoms (anxiety and/or depression). Mean CDIP-58 was 31.9. There was no relationship between faux pas outcomes and motor severity. However, correlation analyses showed that participants who performed worse on the faux pas questionnaire had lower quality of life.

**Conclusion:**

The non-motor symptoms, including social cognition, are often neglected. We have demonstrated that low quality of life in CD is associated with to abnormal social cognition. Clinicians should be mindful of these symptoms, particularly in patients reporting low treatment satisfaction.

## 1 Introduction

Adult-onset idiopathic cervical dystonia (AOICD) has been linked with a variety of cognitive deficits [1]. These form part of the “non-motor” syndrome of AOICD, which includes sensory symptoms (e.g. pain) and psychiatric features like anxiety and depression [2]. A lifetime prevalence of anxiety and depression of up to 70% has been reported [3]. The non-motor symptoms, particularly anxiety and depression, are the main predictors of poor quality of life (QoL) associated with AOICD [4]. The high prevalence of mood disorder in AOICD provide a unique insight into disease pathophysiology and these symptoms frequently pre-date the motor symptoms, and influence the age at onset of AOICD [5].

Cervical dystonia probably is autosomal dominantly inherited with reduced penetrance. [6] Large genetic studies have been inconclusive [7], [8] but it is likely that there are polygenetic changes with environmental influence [9]. Recently it was shown that cells enriched for dystonia related genes also enriched for heritability of neuropsychiatric disorders suggesting a shared genetic origin. [10] Although this link has not been made for AOICD specifically, it is possible, given the high prevalence of anxiety and depression that similar mechanisms exist.

Social cognition refers to the processing of our interactions or behaviours directed towards other people. Many neurodevelopmental, neurological and psychiatric disorders present with social cognitive deficits e.g. Autism Spectrum Disorder, frontotemporal dementia, Alzheimer’s Disease and acquired brain injuries [11]. Theory of mind (ToM), an important feature of social cognition, refers to the ability to attribute mental states to others (also called mentalising) [12]. Functional MRI studies have demonstrated that the medial prefrontal cortex and superior temporal sulcus may mediate aspects social cognition, including ToM [13]. In epilepsy and Alzheimer’s Disease, social cognition deficits are associated with lower QoL [14], [15].

Further clarity about cognitive changes and social cognition in AOICD might open new avenues for treatment, improving QoL and pathophysiological modelling of AOICD. In AOICD, studies into social cognition have shown conflicting findings [16], [17], [18], [19]. Recently largely intact social cognitive abilities were demonstrated in a CD population but facial expressions of fear were not well recognised [19]. Abnormal recognition of disgust has been noted in CD and blepharospasm [20]. However, phenotypic heterogeneity, differences in assessment tools and relatively small study populations make generalisations across AOICD difficult.

We examined social cognition in AOICD patients using the *Faux Pas* (FP) measure [21], which identifies deficits in aspects of social inference (the ‘faux pas’), a component of ToM. We also reviewed the impact of social cognition changes on motor severity, mood, disease disability and QoL.

## 2 Patients & methods

### 2.1 Participants

This was a cross-sectional study. We recruited a convenience sample of 32 patients with isolated AOICD from a single dystonia service. All patients were receiving 3-monthly botulinum toxin (BoNT) injections as the primary treatment. Patients with medications that can impact cognition (anti-cholinergics/ benzodiazepines) and those with severe cognitive impairment were excluded. Demographics and clinical data were also collected.

Motor and non-motor symptoms were rated independently. Dystonia specific assessments were performed by a neurologist. Social cognition measures were administered by a trained psychology research assistant. Assessments were undertaken on the day of clinic attendance for botulinum toxin injection (week 0 of 3-monthly cycle), immediately after injections by the treating neurologist and assessment of motor symptoms, to minimise impact of treatment on outcomes. Assessments were performed together at a single time point.

All participants provided informed consent prior to inclusion and this study was approved by the local research and ethics committee in St Vincent’s University Hospital.

## 3 Methods

### 3.1 Study instruments

1. **Social Cognition**: Faux Pas (FP) measure to assess ToM. This measure assesses patients’ ability to identify faux pas correctly and identifying non-faux pas appropriately across 20 stories [22]. Higher FP ‘correct hits’ and ‘correct rejects’ values indicate better social cognitive performance. Control values and scoring method were adapted from Gregory et al [23].
2. **Mood assessment**: Hospital Anxiety and Depression Scale- Anxiety/ Depression [24] (HADS-A, HADS-D) with a cut-off of ≥ 8 to indicate presence of clinically elevated mood symptoms [25]. The Beck Anxiety Inventory (BAI) and Beck Depression Index-II (BDI-II) with cut-off score of > 13 were also used [26], [27]. HADS is not affected by overlapping physical symptoms but is very brief, whereas Beck measures are more comprehensive but can be artificially elevated by physical symptoms. We used both to best identify mood disturbance.
3. **Motor severity, disability and pain**: The Toronto Western Spasmodic Torticollis Rating Scale-2 (TWSTRS2, cervical dystonia specific instrument) subdomains for severity (TWSTRS2S), disability (TWSTRS2D) and pain (TWSTRS2P) were employed [28].
4. **Quality of life**: The Cervical Dystonia Impact Profile-58 (CDIP58) has been shown to be reliable and validated measure of QoL and measures 7 domains (total, head and neck, pain, sleep, psychosocial, upper limb, walking, mood and, annoyance) [29], [30]. A lower CDIP-58 score indicates better QoL.

Control results for comparison were taken from published data from authors involved in the design of the Faux Pas Test. [23].

### 3.2 Statistical methods

Scores for the FP test were normalised as recommended by the test creators and compared to normative data from the literature [23]. Scores were transformed to z scores to describe the effect size. Correlation analysis using Pearson’s *r* was performed to assess the link between social cognition, mood scores, motor severity and disability and QoL. Further subgroup analysis was performed for patients that met criteria for prominent mood symptoms on any of the 4 mood questionnaires used. Separate correlation analysis was also performed on the data from this group of people with clinically elevated mood symptoms.

## 4 Results

32 participants (19 women) with AOICD were included in this study. Mean age was 57.7-years (±10.1) and mean age at onset was 40.7 years. Participants had a mean of 16.9 years in formal education. Demographics and mean results from the study instruments are listed in Table 1. All participants were receiving BoNT therapy as the primary treatment.

**Table 1:** Demographics and mean score and range across mood measures, social cognitive measures, QoL, and TWSTRS2. *BAI = Beck Anxiety Inventory, BDI = Beck Depression Index, HADS-A = Hospital Anxiety and Depression Scale-* Anxiety*, HADS-D = Hospital Anxiety and Depression- Depression, TWSTRS2S = TWSTRS2 severity, TWSTRS2P = TWSTRS2 pain, TWSTRS2D = TWSTRS2 disability.* Not all patients undertook every test, specific *N* values are provided.

**Variable**	**N**	**Mean (SD)**	**Range**
Age	All participants (32)	57.7 (10.1)	33–73
	Male (13)	53.1 (9.9)	35–72
	Female (19)	59.2 (9.7)	33–73
Age at onset	32	40.7 (10.9)	20–63
Years of formal education	32	16.9 (3.9)	10–24
BAI	29	9.2 (6.8)	0–31
BAI > 13	6 (19%)		
BDI	29	10.2 (10.4)	0–39
BDI > 13	8 (25%)		
HADS-A	19	8.1 (4.5)	1–17
HADS-A ≥ 8	13 (41%)		
HADS-D	26	4.9 (3.7)	0–15
HADS-D ≥ 8	5 (16%)		
CDIP-58 Total	31	31.9 (20.8)	5–83
TWSTRS2S	29	10.6	0–19
TWSTRS2P	29	12	0–25
TWSTRS2D	29	5.3	1–16
		**Mean Z score**	
Faux Pas “correct hits”		**−**0.7	−1 to 1
Faux Pas “correct rejects”		−1.0	−1 to 1

Six (19%) participants had BAI scores > 13, and 8 (25%) participants met criteria for elevated depressive symptoms on the BDI. 13 (41%) participants met criteria for significant mood symptoms on the HADS-A and 20 (63%) participants on the HADS-D.

Patient participants’ performance on the Faux Pas was compared with control group data [23]. With patient participants, mean correct hits on the FP was 0.88 (±0.18) and mean correct rejects was 0.89 (±0.19). The age-matched control group had mean correct hits of 0.95 (±0.1) and correct rejects of 0.99 (±0.1). Our participants had a mean z score of −0.7 for correct hits and z score of −1.0 for correct rejects.

**Correlation analysis:** We performed Pearson’s correlation analysis including outcomes of the FP test as it relates to symptoms of severity (TWSTRS2), quality of life (CDIP-58) and mood scores. The FP ‘correct rejects’ correlated negatively with the BAI (r = -0.387, p = 0.038), such that lower anxiety symptoms were associated with greater accuracy on the social cognition measure but correlations with BDI, HADS-A and HADS-D did not meet significance. The BAI, HADS-A and HADS-D did not correlate significantly with any of the TWSTRS2 domain; the BDI had a moderate correlation with TWSTRS2D (*r* = 0.405, p = 0.29), such that higher symptoms of depression were associated with greater dystonia related disability.

No correlation between FP and any of the TWSTRS2 domains met statistical significance. FP ‘correct hits’ had a moderate negative correlation with quality of life (CDIP58 total), such that a better social cognition performance correlates with better QoL. A similar relationship was also seen between faux pas correct hits which had a moderate negative correlation with CDIP58 psychosocial and CDIP58 annoyance, that is, better performance on FP correct hits correlated with better psychosocial and annoyance on CDIP58. A strong negative correlation was seen between FP correct hits and the CDIP58 walking domain- better social cognitive performance correlated with better quality of life. Table 2 shows specific values of Pearson’s *r* correlation between FP outcomes and CDIP-58 subdomains.

**Table 2:** Results of Pearson’s correlation *r* values with FP outcomes. *NS = not statistically* significant.

	**Faux Pas** Correct Hits	**Faux Pas** Correct Rejects
		
**CDIP58Total**	−0.491**	0.393*
**CDIP58 Mood**	−0.319 (NS)	0.255 (NS)
**CDIP58 Psychosocial**	−0.441*	0.161 (NS)
**CDIP58 Head and neck**	−0.307 (NS)	−0.392*
**CDIP58 Walking**	−0.610**	0.378*
**CDIP58 Annoyance**	−0.428*	0.337 (NS)
**CDIP58 Upper Limb**	−0.311 (NS)	0.361 (NS)
**CDIP58 Sleep**	−0.336 (NS)	0.263 (NS)

* p < 0.05.

** p < 0.01.

Our results, where significant, tend towards a negative correlation between CDIP58 domains and FP outcomes, suggesting that worse social cognitive performance correlates with worse QoL.

**Sub-group analysis of participants with elevated mood scores**: 20 participants (13 women) met criteria for elevated mood symptoms on *at least one* of our four mood rating scales (BAI, BDI, HADS-A or HADS-D). The mean age for this group with clinically elevated mood symptoms was 56.8 years. Mean TWSTRS2S was 10.1, TWSTRS2P was 11.7 and TWSTRS2D was 5.3. A correlation analysis showed that for these patients, FP ‘correct hits’ had a moderate negative correlation with CDIP walking (*r* = -0.545, p = 0.013), such that a better social cognitive performance as measured by FP correct hits was associated with improved performance on the CDIP-58 walking domain.

Walking is an important QoL measure and often exacerbates dystonic symptoms in AOICD [31]. There was no other correlation between FP outcomes and CDIP measures, or any of other assessment tools used.

## 5 Discussion

Here we demonstrate that, based on the FP measure of social cognition, patients with AOICD score in the below average range for z scores for both ‘correct hits’ and ‘correct rejects’, indicating worse social cognition compared with published normative values. Further we demonstrate, for what we believe to be the first time, that social cognitive deficits in AOICD correlate with lower QoL. This is an important consideration in treating patients with AOICD**.** The primary treatment method remains BoNT, but dissatisfaction is high [32]. Cognitive changes can often be overlooked but may prove to be a useful target in improving treatment outcomes and QOL. Although our results do not imply a direct causation, other non-motor symptoms have been linked with lower quality of life. [33].

Our study measures the “theory of mind” aspect of social cognition. One large study reviewing all domains of social cognition in AOICD noted isolated deficits in recognising “fear” as an emotion [19]. An earlier study showed difficulty in recognising “disgust”, whereas recognition of fear was intact [20]. A previous assessment of faux pas using the Faux Pas Recognition Test (our faux pas test is a similar measure) showed that patients with cervical dystonia performed significantly worse on faux pas recognition, compared to controls. They also had difficulty in the interpretation of minor misunderstandings (stories without a faux pas), that is, patients perceived a faux pas where there was none [18]. This inability to comprehend social situations (ToM processing) has the potential to impact social relationships- humans are a uniquely social species and our evolutionary success may have depended on it [34].

A recent artificial intelligence based MRI brain analysis of patients with AOICD and other focal dystonias demonstrated several anatomically and diagnostically distinct regions associated with sensorimotor processing and motor control [35]. Functional MRI studies have demonstrated functional changes compared to controls, in areas relating to head motor control and alterations in basal ganglia connectivity [36], [37] and also cerebellar-cortical connectivity changes in response to BoNT treatment [38]. Social cognitive changes raise more pathophysiological questions. It is possible that functional inputs and connectivity can identify “networks” of areas that could explain AOICD- lesion mapping and patients with acquired CD have been studied for this purpose [39]. Some of the regions implicated in these studies are associated with recognition of facial expressions (an important component of social cognition) e.g. the basal ganglia [40] and the inferior temporal gyrus [41].

Given the prominent non-motor syndrome (of which social cognition is part), any mechanistic explanation for AOICD must also explain the non-motor symptoms. One previous model proposed that the concept of a “neural integrator” of head movements in the region of the Interstitial Nucleus of Cajal can explain some motor symptoms in cervical dystonia [42]. The same article does not provide detail on the various sensory, cognitive and psychiatric features of AOICD. We have previously suggested that a network involving the superior colliculus, amygdala and pulvinar nucleus can account for the motor and non-motor symptoms. However, this is an area that needs further work [43]. Our model is not a contradiction to the neural integrator model but complements it.

Our study has limitations similar to others in AOICD including the small size of the study population. However, this can be mitigated to some extent, as in our case, by careful selection of homogenous populations and comparisons with appropriate control data. Another difficulty lies in selecting suitable cognitive tests. Studies have demonstrated varying changes in social cognition, and this may be due to differences in assessment method and the many aspects of social cognition, i.e., social cognition is not one entity[44]. As no “gold standard” exists for social cognitive measurements, future research in clinical social neuroscience should focus on sensitive and specific assessment of social cognition in clinical populations. Recruitment of larger populations and studies sufficiently powered to include the different AOICD phenotypes and the various forms of focal dystonias are also required.

Clinicians could consider integration of neuropsychology and other multi-disciplinary services into dystonia clinics. These can provide assessments and interventions that may improve treatment satisfaction and outcomes and, ultimately, patient QOL.

## Funding sources and conflict of interest

The authors declare that there are no conflicts of interest relevant to this work.

## Financial disclosures for the previous 12 months

The authors declare that there are no additional disclosures to report.

## Ethical compliance statement

This study was approved by the research and ethics committee at St Vincent’s University Hospital. All participants provided informed consent.

We confirm that we have read the Journal’s position on issues involved in ethical publication and affirm that this work is consistent with those guidelines.

## Declaration of Competing Interest

The authors declare that they have no known competing financial interests or personal relationships that could have appeared to influence the work reported in this paper.
